# Author Correction: Genome assembly and annotation of *Meloidogyne enterolobii*, an emerging parthenogenetic root-knot nematode

**DOI:** 10.1038/s41597-020-00747-0

**Published:** 2020-11-19

**Authors:** Georgios D. Koutsovoulos, Marine Poullet, Abdelnaser Elashry, Djampa K. L. Kozlowski, Erika Sallet, Martine Da Rocha, Laetitia Perfus-Barbeoch, Cristina Martin-Jimenez, Juerg Ernst Frey, Christian H. Ahrens, Sebastian Kiewnick, Etienne G. J. Danchin

**Affiliations:** 1 Université Côte d’Azur, IN RAE, CNRS, Institut Sophia Agrobiotech, Sophia Antipolis, France; 2 Strube Research GmbH & Co. KG, Hauptstraße 1, 38387 Söllingen, Germany; 3 Laboratoire des Interactions Plantes-Microorganismes, INRAE, CNRS, 31326 Castanet-Tolosan, France; 4 Agroscope, Molecular Diagnostics, Genomics & Bioinformatics, Wädenswil, Switzerland; 5 Swiss Institute of Bioinformatics, Bioinformatics, Wädenswil, Switzerland; 6 Julius Kühn Institute (JKI) - Federal Research Centre for Cultivated Plants Federal Research Centre for cultivated Plants, Messeweg 11/12, 38104 Braunschweig, Germany

Correction to: *Scientific Data* 10.1038/s41597-020-00666-0, published online 05 October 2020

Following publication of this Data Descriptor, it was found that a number of references were incorrectly cited in the ‘Species/Feature’ column of Table 3 and a single reference was incorrectly cited in the main-text paragraph titled ‘Divergence level between duplicated genome blocks’. These errors have been corrected and references 70–74 have been added to both the HTML and PDF versions of this publication.

